# Reminiscenses by M. A. Taylor, M. D.

**Published:** 1914-07

**Authors:** 


For Texas Medical Journal.

### Reminiscenses by M. A. Taylor, M. D.

In the following sketch by the late Dr. M. A. Taylor, of Austin, will be found an account of the organization of the Texas State Medical Association, and something of the difficulties which the doctors of those days encountered. To these grand pioneers much credit is due, and the Texas physicians who enjoy the luxury of good roads and an up-to-date automobile should not forget them.

The Austin physicians feeling the necessity of an organization, we therefore organized in October, 1854, and named our association "The Travis County Medical Society." The officers elected at the first meeting were Dr. W. A. Morris, President; Dr. J. M. Litten, Vice-President; Dr. R. N. Lane, Secretary, and Dr. M. A. Taylor, Treasurer.

The general object of the association was to bring about closer and more friendly relations between the practicing physicians of our neighborhood.

Our society adopted a constitution and by-laws for its government, fixing the time for its monthly meetings, at which time it was the custom for its members to read "papers" and to report interesting and unusual cases, and to discuss methods and means for advancing in our chosen profession.

By reason of our small membership we were enable to meet promptly, and frequently long discussions and timely dissertations engaged our attention from its organization up to 1856. We then deemed it wise and practical to organize a larger society of medical men. So we organized the Texas State Medical Association. Dr. George W. Cupples, a graduate of Edinburgh, Scotland, was elected as our President; Dr. W. A. Morris, First Vice-President; Dr. J. M. Litten, Second Vice-President; Dr. R. N. Lane, Secretary, and Dr. M. A. Taylor, Treasurer. We had the following named physicians enrolled as members: Drs. Morris, Litten, Lane, Baker and Taylor, of Austin, and Dr. George W. Cupples and Dr. F. Herff, of San Antonio; Dr. W. C. Dixon and Dr. Kitterell, of Grimes county, and Dr. Sayers, of Bastrop.

We held our meetings in the old Capitol building, which then occupied the site that is now used for our new city hall. The first meeting was a great success, as far as the discussions and papers presented thereat. We local physicians tendered a complimentary banquet to our visiting brethern. There was but one drawback to that festive occasion, and that was that our guests were too few in number.

In October, 1857, we held the second annual meeting of our Texas State Medical Association. Dr. W. C. Phillips was elected President. I remember that his annual address was a fine paper on "Medicine and Its Relations to the People."

When the time for our third meeting came around, we found it impracticable to continue the State Association any longer, from the fact that transportation was so difficult that the physicians of our State could not attend the annual meetings at Austin, or any other place, as the means of travel was limited to horseback in most cases, and private conveyances over the most favored routes. Our highways were practically what nature had made them, and there were but few roads in the country, and no bridges at all. Nevertheless, to our credit, be it said, we Austin physicians kept up the active work of our Travis County Medical Society until the close of the late war, when our organization practically existed in name only. But feeling the importance of keeping it alive, we held irregular meetings, there discussing the various possibilities confronting us, and the probable outcome of the Southern States and the success of its people.

Hope was often brightened by the news of the times; then, again, the clouds of war and mists of uncertainty would mar our hopes for the future. So, five years passed, and, finally, General Lee's surrender took place, and the news came as a shock to our people, that cast a pall over our State. Then followed the tedious days of reconstruction, and the assassination of President Abraham Lincoln.

Andrew Johnson succeeded to the Presidency of the United States. Hopes for better conditions under his administration brought smiles to the Southern people. But, alas, their hopes were soon shattered, as Andrew Johnson's policy was not satisfactory to the Northern people, and, following upon his failure to accomplish the changes wanted by the South, he soon lost favor with the people of the former Confederate States. Such, differences of opinion seriously affected the success of our people, and left us for a long time in doubt as to the future.

Before the arrival of the Federal forces, many of our Southern men felt it best for them to seek protection in other lands. Some in Europe and some in Old Mexico. They left their families to the care of friends who remained in Austin.

I mention these facts to show the unsettled condition of those times and the anxiety of our people, and thus explain the irregularity of the meetings of our Travis County Medical Society.

The unsettled conditions of our government had its serious effect on the lives and health of our people. A greater amount of charity work had to be done, and the practicing physicians of this community had their proportion of the responsibility, labor and expense, caused by such conditions; and right nobly did they meet and perform their duty to the poor and needy.

The lack of proper cultivation of our productive farms, and absence of proper care of, and attention to, the ranch lands, coupled with the previous great waste and loss of personal property, led to great mental and physical suffering among our home people.

But finally the war clouds rolled away, and the "dove of peace" settled over our bounteous and sun-kissed land.

With fortunes shattered, if not completely lost, our people awoke to the call of returning prosperity, and began to gather up the fragments of what was left them.

The change for the worse was great in our home laborers and the comforts associated therewith. The emancipation of five million of our slaves left many of our best families almost helpless for lack of servants in their homes and on their farms.

Such was the result caused by the shock of arms and the consequent paralysis experienced by the Southern people as an aftermath of that long and terrible war.

Blessed be those whose largeness of heart and love for their fellow man caused them to render aid to the helpless and dependent ones here in Austin in those days that tried men's souls.

A very few of those splendid men are with us now, but the most of them have gone to their final reward, where love will reign forever and forever more.

Austin, Texas, September, 1908.

